# Atomic Force Microscopy to Characterize Antimicrobial Peptide-Induced Defects in Model Supported Lipid Bilayers

**DOI:** 10.3390/microorganisms9091975

**Published:** 2021-09-17

**Authors:** Kathleen W. Swana, Ramanathan Nagarajan, Terri A. Camesano

**Affiliations:** 1 Department of Chemical Engineering, Worcester Polytechnic Institute, Worcester, MA 01609, USA; kathleen.w.swana.civ@army.mil; 2 U.S. Army Combat Capabilities Development Command Soldier Center, Natick, MA 01760, USA; ramanathan.nagarajan.civ@army.mil

**Keywords:** antimicrobial peptide, atomic force microscopy, lipid bilayer, membrane, antimicrobial, pore formation

## Abstract

Antimicrobial peptides (AMPs) interact with bacterial cell membranes through a variety of mechanisms, causing changes extending from nanopore formation to microscale membrane lysis, eventually leading to cell death. Several AMPs also disrupt mammalian cell membranes, despite their significantly different lipid composition and such collateral hemolytic damage hinders the potential therapeutic applicability of the AMP as an anti-microbial. Elucidating the mechanisms underlying the AMP–membrane interactions is challenging due to the variations in the chemical and structural features of the AMPs, the complex compositional variations of cell membranes and the inadequacy of any single experimental technique to comprehensively probe them. (1) Background: Atomic Force Microscopy (AFM) imaging can be used in combination with other techniques to help understand how AMPs alter the orientation and structural organization of the molecules within cell membranes exposed to AMPs. The structure, size, net charge, hydrophobicity and amphipathicity of the AMPs affect how they interact with cell membranes of differing lipid compositions. (2) Methods: Our study examined two different types of AMPs, a 20-amino acid, neutral, α-helical (amphipathic) peptide, alamethicin, and a 13-amino acid, non-α-helical cationic peptide, indolicidin (which intramolecularly folds, creating a hydrophobic core), for their interactions with supported lipid bilayers (SLBs). Robust SLB model membranes on quartz supports, incorporating predominantly anionic lipids representative of bacterial cells, are currently not available and remain to be developed. Therefore, the SLBs of zwitterionic egg phosphatidylcholine (PC), which represents the composition of a mammalian cell membrane, was utilized as the model membrane. This also allows for a comparison with the results obtained from the Quartz Crystal Microbalance with Dissipation (QCM-D) experiments conducted for these peptides interacting with the same zwitterionic SLBs. Further, in the case of alamethicin, because of its neutrality, the lipid charge may be less relevant for understanding its membrane interactions. (3) Results: Using AFM imaging and roughness analysis, we found that alamethicin produced large, unstable defects in the membrane at 5 µM concentrations, and completely removed the bilayer at 10 µM. Indolicidin produced smaller holes in the bilayer at 5 and 10 µM, although they were able to fill in over time. The root-mean-square (RMS) roughness values for the images showed that the surface roughness caused by visible defects peaked after peptide injection and gradually decreased over time. (4) Conclusions: AFM is useful for helping to uncover the dynamic interactions between different AMPs and cell membranes, which can facilitate the selection and design of more efficient AMPs for use in therapeutics and antimicrobial applications.

## 1. Introduction

Due to the escalating occurrence of bacterial resistance to antibiotics in the past several decades, there has been growing interest in the identification and development of antibiotic alternatives, such as antimicrobial peptides (AMPs). While hundreds of AMPs have been identified in nature and many more have been synthetically produced, only 36 are under pre-clinical or clinical trial review right now, due to issues with stability and bioavailability [1]. AMPs are part of the innate immune systems of most eukaryotic organisms and are thought to kill pathogens through cell membrane disruption, but the active mechanism behind the membrane destabilization is not well understood and can vary depending on the physicochemical properties and biochemical nature of the AMP, as well as the different types of cell membranes. Many AMPs are cationic and are believed to initially associate with the anionic peptidoglycan layer of Gram-positive bacterial cell walls or the lipopolysaccharide layers on the outer membrane of the Gram-negative bacteria, before interacting with the cytoplasmic membrane of these bacterial cells composed dominantly of anionic lipids. There are wide variations possible in the AMP charge, including low or zero values, and therefore, it is recognized that other characteristics such as the hydrophobicity of the AMP and the type of secondary structure they form in solution are also critical in determining AMP–membrane interactions.

Techniques such as oriented circular dichroism (OCD), nuclear magnetic resonance (NMR) spectroscopy and quartz crystal microbalance with dissipation monitoring (QCM-D) have been employed to probe the interactions of AMPs with membrane models such as monolayers, micelles, liposomes, multilayers and supported lipid bilayers (SLBs) [2,3,4,5,6,7,8]. In conducting these experiments, the AMP has been either incorporated as a static part of the membrane or it is allowed to dynamically encounter the membrane. For example, in OCD experiments, AMP-containing multilayers are preformed, and their properties are investigated. In contrast, in QCM-D experiments, the AMP-free SLBs are formed first and then AMP is allowed to dynamically interact with the membrane. These experiments have shown that peptides can exhibit distinct states once they are associated with the lipid bilayers. According to the carpet model, AMPs may adsorb parallel to the bilayer membrane and “carpet” the lipid surface. As the peptide concentration reaches a critical level, the peptides and lipids reorganize to leave the membrane as aggregates or micelles. The barrel-stave model supposes that AMPs may insert perpendicularly into the lipid bilayer and form cylindrical or toroidal pores, through which large molecules can travel, disrupting ion gradients and causing the cell to die.

**Figure 1:**
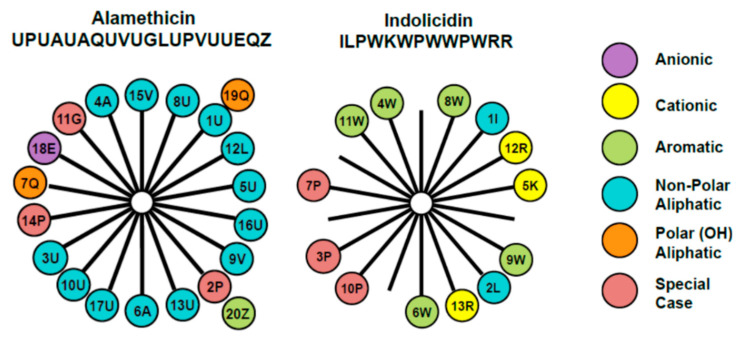
Helical wheel diagrams for alamethicin and indolicidin [7] explored in this study. This diagram provides a projection of amino acids perpendicular to the helix long axis assuming that the peptide exists in an α-helical secondary structure. Since the α-helix contains 3.6 residues per turn, adjacent residues on the peptide are separated by 100° on the helical wheel. The number represents the position of the amino acid in the peptide chain and the letter denotes the commonly accepted single letter notation used to designate amino acids. The uncommon amino acids α-aminoisobutyric acid and l-phenylalaninol in alamethicin’s amino acid sequence are represented by U and Z, respectively. These diagrams reveal differences in the placement of charged amino acid residues, as well as hydrophobic and polar side chains.

The membrane-active antimicrobial peptides alamethicin and indolicidin were chosen for examination in this study due to their different structures and mechanisms of action. The amino acid composition and arrangement within the two peptides are represented through the helical wheel diagram in Figure 1. Alamethicin includes two amino acids, aminoisobutyric acid and L-phenylalaninol, that are rarely found in nature. It contains a negative charge associated with the glutamic acid residue near the C-terminus. However, this side chain is typically protonated when the peptide is oriented in a transmembrane state, making alamethicin’s net charge effectively zero in a peptide–lipid membrane system. The helical wheel diagram shows a clear separation between the dominant hydrophobic face and a smaller polar face in the alpha-helical structure for this peptide.

Alamethicin has been shown to form voltage-gated ion channels in membranes [9,10,11]. The peptide is thought to attach to the lipid headgroups and insert into membranes, forming pores that can contain 3–11 peptide molecules [12,13,14,15,16]. Neutron in-plane scattering experiments have shown that in 1,2-dilauroyl-sn-glycero-3-phosphatidylcholine (DLPC) membranes, these pores exhibit inner and outer diameters of ~1.8 and ~4.0 nm, respectively [17]. QCM-D studies suggested that alamethicin will form cylindrical pores in the supported phosphatidylcholine (PC) membrane [5] and the results were interpreted as indicating pores with diameters of 3.9 nm (if formed by eight peptides) or 4.6 nm (if formed by 10 peptides). QCM-D also showed large changes such as complete bilayer removal at different conditions.

Indolicidin is one of the smallest naturally occurring linear peptides. Its amino acid content is quite remarkable because of the five tryptophan and three proline residues. Indolicidin carries a net charge of + 4 at pH 7 and assumes a specific coiled and folded conformation when in contact with a cell membrane [18]. Intramolecular cation–π electron interactions allow it to assume a folded, boat-shaped conformation with positive charges at the peptide termini and a hydrophobic core. The helical wheel diagram shows a separation of hydrophobic and hydrophilic residues, but the large presence of prolines in this small peptide prevents it from assuming an α-helical secondary structure. Studies have shown that indolicidin does not cause hemolytic lysis at concentrations below 30 μM. QCM-D studies suggest that indolicidin will adsorb to a supported phosphatidylcholine (PC) membrane and partially insert into the bilayer [7]. Other studies, including a combination of AFM and QCM-D, showed that indolicidin interacts mainly on the surface of lipid bilayers, rather than forming true pores [19]. While alamethicin’s α-helicity and amphiphilicity promotes membrane insertion, indolicidin favors surface adsorption over insertion, largely due to its folded structure. However, since indolicidin also contains hydrophobic amino acid residues, it also partially inserts into the membrane.

To analyze the effect of AMPs on membrane structure and stability, we used atomic force microscopy (AFM) imaging to examine AMP-induced changes to supported lipid bilayers (SLBs). AFM is a sensitive technique that uses a sharp-tipped cantilever to scan the surface of samples and produce images with nanoscale resolution. Forces between the tip and sample cause cantilever deflections that are measured using a laser spot reflected into a photodiode array, producing an image reflecting the height variations on the surface. AFM has been used to examine changes in the supported lipid membrane structure and thickness due to exposure to peptides [20,21,22,23,24,25,26,27,28,29,30,31,32,33,34,35], surfactants [36,37] and dendrimers [38]. For instance, Lam et al. used AFM to image the structural transformations in a zwitterionic 1,2-dimyristoyl-sn-glycero-3-phosphocholine (DMPC)-supported lipid membrane exposed to the cationic AMP protegrin-1 and saw evidence of pore formation and wormlike micelles in the membrane [21].

The AFM experiments with alamethicin and indolicidin were conducted using a supported lipid bilayer constructed of zwitterionic phosphatidylcholine (PC). In selecting the lipids for the membrane model, our choice was influenced by the following three considerations: (i) Bacterial membranes have either all anionic lipids (Gram-positive bacteria) or a mixture of anionic and zwitterionic lipids (Gram-negative bacteria). The successful formation of robust and stable SLBs with a large proportion of anionic lipids on quartz surfaces has not yet been achieved. Our laboratory has recently made progress in developing protocols for first treating the quartz surface with cationic moieties and then forming completely anionic SLBs composed of mixtures of phosphatidyl glycerol, PG, and lysophosphatidyl glycerol, LPG, at various compositions. We plan to use such anion dominant membrane models representing bacterial cells in future studies. (ii) Egg PC and other zwitterionic phospholipids, representative of the erythrocyte membrane, have been shown to form highly reproducible supported lipid bilayers (SLBs) and, therefore, all the experiments reported in this paper have used egg PC as the lipid. (iii) Our prior studies on alamethicin and indolicidin using the QCM-D technique have been carried out using SLBs made of egg PC. Therefore, the observations from the present AFM studies can be combined with the observations from the QCM-D studies, to capture various features of AMP–SLB interactions, given that no single method of investigation is adequate to characterize the AMP-SLB systems.

For the AFM experiments, various AMP concentrations were examined, as well as the time progression of AMP-induced membrane destabilization. By inferring information from AFM images, we aim to add on to the understanding of alamethicin– and indolicidin–membrane interactions that were developed in previous QCM-D studies [5,7].

## 2. Materials and Methods

### 2.1. Antimicrobial Peptides

Alamethicin and indolicidin were obtained from Sigma Aldrich (St. Louis, MO, USA) and New England Peptide (Gardner, MA, USA), respectively. All peptides were suspended in Tris-NaCl buffer (100 mM sodium chloride and 10 mM tris (hydroxymethyl) aminomethane, pH 7.8) and stored at −20 °C. A range of AMP concentrations (1, 5 and 10 µM) were tested to examine the concentration dependency of AMP–membrane interactions.

### 2.2. Supported Lipid Bilayer Preparation

Egg phosphatidylcholine (Avanti Polar Lipids, Alabaster, AL, USA) vesicles were made and stored in Tris-Nacl buffer. Egg phosphatidylcholine (PC) lipids dissolved in ethanol were dried under a stream of nitrogen gas and placed in a vacuum desiccator overnight. The dried lipids were reconstituted in Tris-NaCl buffer to create a stock concentration of 2.5 mg/mL. The lipid mixture was then vortexed and homogenized through 5 freeze–thaw cycles.

The egg PC lipids were sonicated with an ultrasonic dismembrator (Model 150T, Fisher Scientific, Waltham, MA, USA) in an ice bath for 30 min in pulsed mode with a 30% duty cycle (3 s on followed by 7 s pause) at an amplitude of 60 to form small unilamellar vesicles (SUVs). Sonicator probe particles were removed from solution through centrifugation (5415D Microcentrifuge, Eppendorf, Hamburg, Germany) at 16,000× *g* for 10 min. The stock solution was diluted using Tris-NaCl buffer to 0.1 mg/mL for each experiment.

Supported egg PC bilayers were formed on muscovite mica (Electron Microscopy Sciences, Hatfield, PA, USA), which was cut into 0.7-centimeter-diameter circular sheets using a 3-hole punch. The mica circles were fixed in a 1.5-centimeter-diameter well in a 1.2-millimeter-thick depression slide using epoxy. Before each experiment, the microscope slides were rinsed with 2% sodium dodecyl sulfate and DI water and dried with nitrogen gas. The mica was cleaved so that the surface was visually smooth and etched with a SPI Plasma Prep II Plasma Etcher (SPI Supplies, West Chester, PA, USA).

### 2.3. Atomic Force Microscopy (AFM)

All samples were scanned in liquid using a Veeco Dimension Icon with ScanAsyst (Bruker, Santa Barbara, CA, USA). A silicon nitride ScanAsyst Fluid+ cantilever with a spring constant of 0.7 N/m and nominal tip radius of 2 nm was used. Images were captured with 512 × 512 resolution at a scan rate of 1 Hz.

After the mica surface was scanned in Tris-NaCl buffer to validate the smoothness of the surface, excess buffer was pipetted from the mica surface and replaced with ~0.1 mL of 0.1 mg/mL egg PC vesicle solution. AFM image scanning began at least 5 min after the introduction of vesicles to allow sufficient time for stable supported lipid bilayer (SLB) formation. Once the presence of a smooth SLB on the mica surface was established, AMPs were introduced into the system. At least 0.25 mL of the experimental concentration of alamethicin or indolicidin was pipetted onto the periphery of the well in 10-microliter increments every 5 s, while simultaneously removing lipid vesicle solution in 10-microliter increments to simulate slow pump flow that was present in our QCM-D experiments. Before bringing the AFM tip to the mica surface in liquid, a drop of AMP solution was placed on the tip, instead of buffer, so that the AMP concentration would not be diluted. The surface was then scanned to image AMP-induced changes to the SLB at a minimum of 3 different locations on the sample.

All images were flattened using the first order polynomial flatten command in the NanoScope Analysis software to delete low frequency noise and remove tilt. Scan lines resulting from noise or skips were also removed from the final images. Roughness values were reported for each 5 µm × 5 µm image as the root mean square (RMS) average of height deviations in the image using the following equation:

(1) $$R_{q}=\sqrt{\frac{\sum^{\;}Z_{i}{}^{2}}{N_{P}}}$$

where *Z_i_* is a specific *Z* (height) value and *N_p_* is the total number of points. Roughness measurements were used as a quantitative method of analyzing changes to the SLB over time.

## 3. Results

### 3.1. Alamethicin Forms Large Defects and Causes Complete Lipid Removal

**Figure 2:**
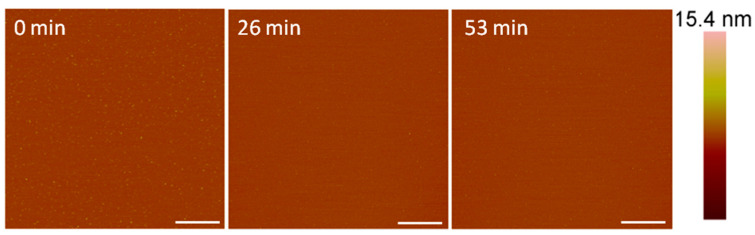
Representative AFM (height) images of a supported PC membrane after exposure to 1 µM alamethicin, with z scale bar shown on the far right. The stable lipid membrane before peptide injection is shown at t = 0 min. The scale bars represent 1 µm.

The AFM imaging of the supported PC bilayer revealed a smooth membrane with small particles on the surface (Figure 2). These particles may have been small lipid aggregates that were present after lipid bilayer formation. Upon injection of 1 µM alamethicin, many of the particles were removed and no visible membrane destabilization took place over the course of 1 h.

**Figure 3:**
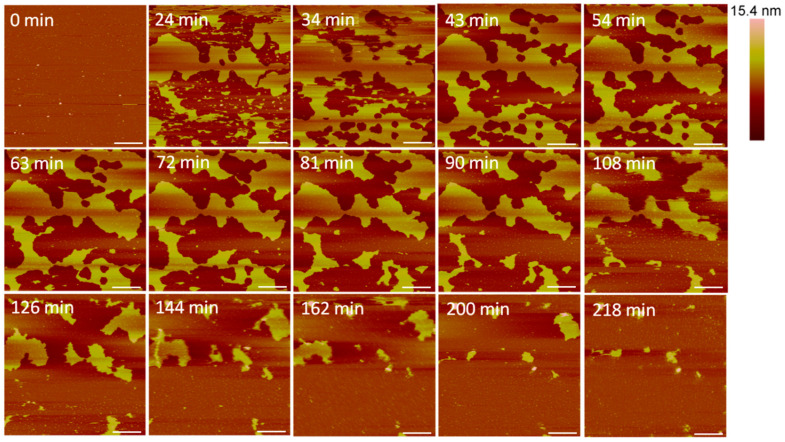
Defects formed in a supported PC bilayer after injection of 5 µM alamethicin over 218 min. Each AFM image was scanned in the same location on the substrate. The scale bars represent a distance of 1 µm.

Large defects, or pores, formed in the lipid membrane upon exposure to 5 µM alamethicin, leaving small islands of lipids amidst larger, connected lipid bilayer patches (Figure 3). Due to the rapid nature of peptide–membrane interactions, we were unable to capture the initial stages of peptide binding and membrane removal. QCM-D studies have shown that the initial peptide binding interactions of alamethicin and indolicidin occur in less than 10 min [7]. High quality imaging of soft systems such as this requires a very slow scan speed, and therefore, we were unable to capture quicker initial reactions with the AFM. Over 3.5 h, lipids continuously shifted around the perimeter of the patches and were removed from the mica surface. Smaller patches of lipids also gradually left the mica surface or combined with neighboring planar lipid patches over time.

**Figure 4:**
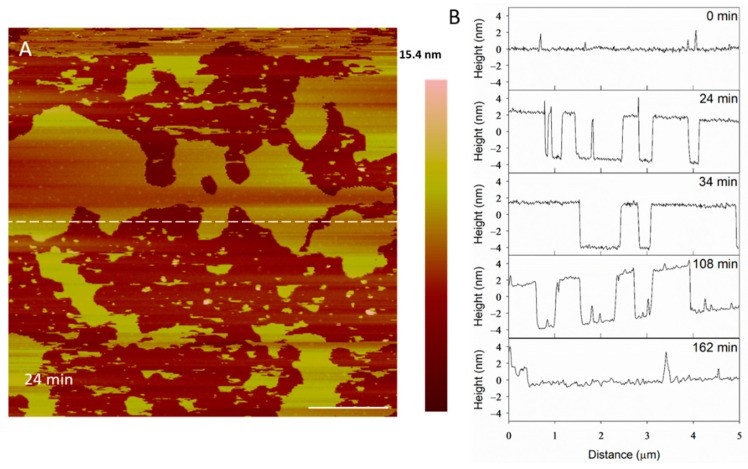
Evolution of membrane defects over time during incubation with 5 µM alamethicin. The dashed line (**A**) shows the location of the height profiles (**B**). The scale bar represents 1 µm.

The height profiles of the membrane at the same location on the substrate (Figure 4A) show some defects widening and others filling in as time progressed, indicating that the lipids at the perimeter of the planar membrane were fluid. Before alamethicin was introduced to the system (t = 0 min), the lipid bilayer was relatively smooth, with 1–2-nanometer particles on the surface (Figure 4B). At 24 min, large membrane defects had formed that were approximately 5 nm deep, which is the expected height of a PC bilayer. In the height profiles, the height of the bilayer surface at 0 min is different than that at 24 min (roughly 2 nm) because AFM does not allow for the direct comparison of heights between images. Therefore, the y-axis is meant to show the depth of the defects, rather than allow for the direct comparison of the height value at a specific position on the substrate. After the membrane had been exposed to the peptide for 34 min, the lipids began to shift and fill in some of the spaces observed in the height profile. The membrane defects expanded, and the lipid material left gaps in the membrane at 108 min. At 162 min, most of the lipids had been removed and the profile shown at a height of 0 nm represents the mica substrate.

**Figure 5:**
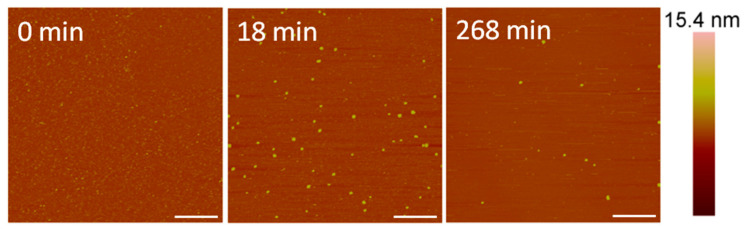
AFM images of the supported PC membrane after exposure to 10 µM alamethicin. The scale bars represent a distance of 1 µm.

At a concentration of 10 µM, alamethicin appeared to remove the bilayer entirely before the first image was taken at 18 min (Figure 5). We were unable to capture an image of earlier time points due to the time needed to reposition the AFM tip on the sample surface and complete the initial scan. Therefore, any action between the peptides and lipid membrane that occurred in the first several minutes could not be captured using AFM.

Although differentiating between an SLB and the mica substrate is difficult in these images, the particles (<10 nm in height) visible on the substrate surface after 18 min of AMP exposure (Figure 5) offer clues about the nature of the surface. Since a large membrane defect formation was observed with an alamethicin concentration of 5 µM, we expected to see defects or bilayer patches remaining on the mica surface if the lipid bilayer was still supported on the mica. These particles, which are likely lipid or peptide–lipid aggregates, appear to be all that remained of the lipid bilayer on the mica surface, however. Over the course of 4 h, some aggregates attached to the surface and most of the original particles present at 18 min gradually left the mica surface. The scanning motion of the AFM tip may have also contributed to the removal of these particles.

**Figure 6:**
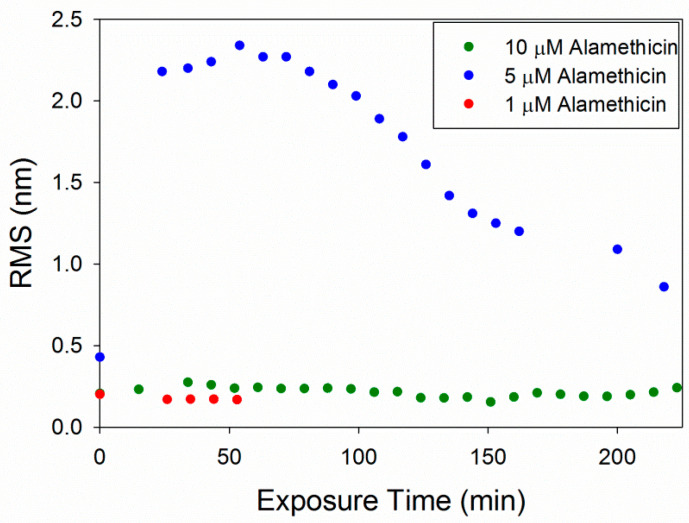
Root mean square (RMS) roughness values calculated for each 5 µm × 5 µm image of the supported PC bilayer after exposure to various alamethicin concentrations.

The root mean square (RMS) roughness values were calculated to provide a quantitative method of analyzing changes in the membrane over time (Figure 6). As observed in the AFM images, 1 µM alamethicin did not change the surface of the membrane over ~1 h and, therefore, the roughness did not change. At 5 µM, alamethicin substantially increased the roughness of the membrane surface within 24 min. The roughness peaked at 54 min and gradually decreased over the next 2.5 h. This decrease in roughness corresponds with the loss of small islands of lipid on the mica surface and around the bilayer patches, revealing more of the smooth mica surface. The complete removal of lipids at 10 µM alamethicin produced lower roughness values that remained constant over 4 h. The RMS values for 10 µM alamethicin were higher than those for 1 µM concentrations, however, presumably due to the presence of aggregates left on the surface of the mica.

The concentration-dependent membrane disruption that was observed as a result of alamethicin exposure is consistent with a previous QCM-D study on PC membranes [5]. Monitoring the changes in the mass and rigidity of a supported PC membrane formed on silica did not show substantial changes to the lipid bilayer when exposed to 1 µM alamethicin in QCM-D experiments. AFM imaging of the membrane after injection of 1 µM alamethicin also did not show visible membrane disruption (Figure 2).

The large defect formations observed in the membrane after exposure to 5 µM alamethicin (Figure 3) agreed with the QCM-D results showing decreases in mass at all depths of the SLB after 1 h of incubation with the same peptide concentration [5]. In the QCM-D experiments, the lipid membrane lost roughly 15–20% of its original mass. From visual analysis, more than 15–20% of the lipid bilayer appeared to be removed in the AFM images after 1 h, but this lipid removal could have been due to forces resulting from the motion of the cantilever. Previous studies have shown that the scanning AFM tip can affect the position of particles on a surface, resulting in the movement of weakly adsorbed peptide aggregates on a bilayer or even the rupture of surface-adsorbed lipid vesicles [22,39]. Although the QCM-D results indicated that alamethicin likely lined the pores in the membrane, we were unable to distinguish individual peptide molecules in our AFM images due to the size. Alamethicin monomers can be approximated as cylinders 3.2 nm long and 1.1 nm wide [16,40] and they may not be distinguishable from the adjacent 5-nanometer-tall lipid bilayer when scanning with a 2-nanometer-radius tip. In a recent study, Abbasi et al. [41] obtained high resolution AFM images of pre-formed bilayers incorporating alamethicin (at a peptide to lipid ratio of 0.1) on a gold surface, using an equimolar zwitterionic 1,2-dimyristoyl-sn-glycero-3-phosphocholine (DMPC) and anionic 1,2-dimyristoyl-sn-glycero-3-phosphorylglycerol (DMPG) lipid mixture and also an equimolar lipid mixture of zwitterionic DMPC with anionic egg PG. They interpreted the AFM images as indicating clusters of alamethicin-induced pores in the bilayer, with a pore diameter of 2.3 ± 0.3 nm for the DMPC/DMPG bilayer and 2.0 ± 0.2 nm for the DMPC/egg-PG bilayer [41]. Their conclusion was based on high resolution AFM images and measuring the lateral distances between contrasting phase spaces. Since AFM provides only accurate depth profiles, it is not clear whether one can interpret the lateral distances estimated from the images as the pore diameter.

The membrane changes induced by 10 µM alamethicin observed using AFM (Figure 5) differed somewhat from the membrane disruption inferred from the QCM-D experiments [5]. Although near-complete bilayer removal was observed in the AFM images, only about 20% of the original bilayer mass was removed from a silica surface in a similar QCM-D experiment. These variations were likely due to differences in the experimental setup of the two techniques. First, the QCM-D measurements were performed in a flow system, whereas the AFM images were scanned in a static system. The scanning motion of the AFM tip also may have caused more lipid removal than in an undisturbed lipid membrane. Furthermore, variations in the amount of lipid removal observed may have been due to differences in the substrates used as lipid bilayer supports. In the QCM-D experiments, a silica-coated quartz sensor was used as the substrate, while the AFM images were scanned on a mica substrate. Mica was chosen as the substrate for the AFM experiments because its cleaved surface is smoother than that of the silica QCM-D sensor. The QCM-D sensor surface exhibits height variations ranging across several nanometers, making the identification of lipid membrane defects difficult on these substrates. Although mica is mainly composed of silicate minerals, which are related to silica, differences in the surface roughness and composition may have impacted the retention of lipid molecules on the surface. A previous study by Benes et al. found that supported 1,2-dioleoyl-sn-glycero-3-phosphocholine (DOPC) and DOPC/1,2-dioleoyl-sn-glycero-3-phospho-L-serine (DOPS) membranes formed and behaved differently on mica and silica surfaces [42]. The membranes were less stable in the Tris-HCl buffer than in the HEPES buffer when formed on mica, but the buffer did not influence membrane stability when supported on silica. These differences in membrane behavior suggest that the two surfaces exhibit different properties that can affect their interactions with lipids.

The loss of lipid mass from the membrane due to alamethicin action was also observed in a study by Oliynyk et al. on 1,2-dipalmitoyl-sn-glycero-3-phosphatidylcholine (DPPC)-supported membranes [26]. Circular and elongated membrane defects were revealed using AFM imaging of DPPC membranes with 1 and 4 mol% alamethicin that were formed by the direct fusion of lipid–peptide small unilamellar vesicles (SUV) on mica. Less mass loss and smaller membrane defects were observed than in our study. The results of the two studies could not be directly compared, however, since the peptide concentrations in our experiments were bulk concentrations and the ratio of peptide to lipid in our membranes is not known. Additionally, although DPPC is a major component of the lipid mixture that is included in egg PC, egg PC also contains 1,2-distearoyl-sn-glycero-3-phosphocholine (DSPC) and other phospholipids that may influence their interactions with the peptides.

### 3.2. Indolicidin Forms Smaller, Unstable Holes in the Membrane

**Figure 7:**
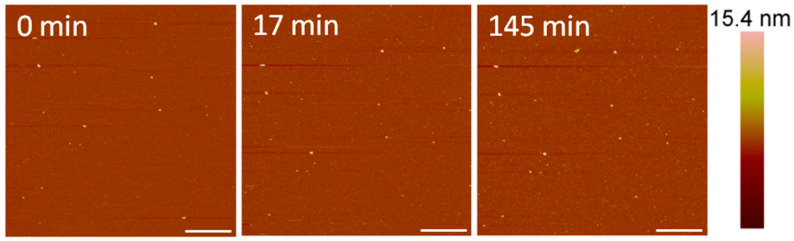
AFM image of a supported PC membrane exposed to 1 µM indolicidin. The scale bars represent a distance of 1 µm.

After exposure to 1 µM indolicidin, visible changes to the membrane were not observed (Figure 7). Before peptide was added, the PC membrane surface contained occasional small particles (~40–50 nm in height) that may have been vesicles. These particles remained after scanning for 2.5 h, although other particles, possibly peptides or peptide–lipid aggregates, were observed to attach to the membrane. Peptide–lipid aggregates may have formed in the solution if indolicidin attached to the membrane and displaced a small amount of lipids, resulting in defects that were too small to be resolved by the 2-nanometer AFM tip radius. Membrane-bound vesicles were also observed in an AFM study by Oreopoulos et al. examining indolicidin action on a DOPC:DSPC:cholesterol membrane [27]. These vesicles were hypothesized to be lipids displaced from the membrane by indolicidin action.

**Figure 8:**
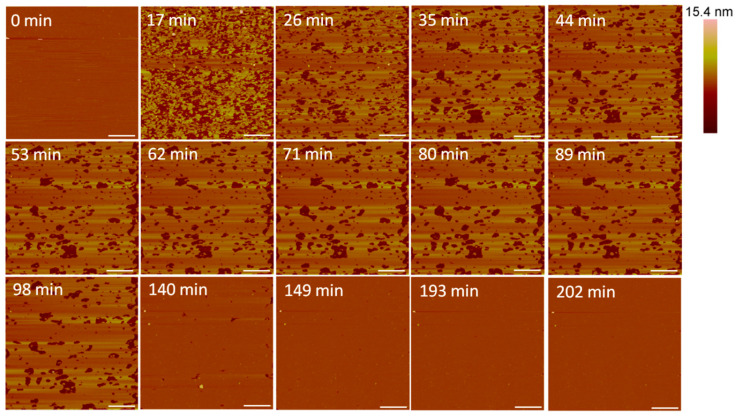
Defects formed in a supported PC membrane after injection of 5 µM indolicidin. The scale bars represent a distance of 1 µm.

At 5 µM, indolicidin formed defects in the membrane (Figure 8) that were smaller than those created by alamethicin at the same concentration (Figure 3). Within 26 min, many of the defects appeared to reorganize and consolidate to make the surface less rough. Over ~ 3 h, these defects filled in to form a smooth membrane.

**Figure 9:**
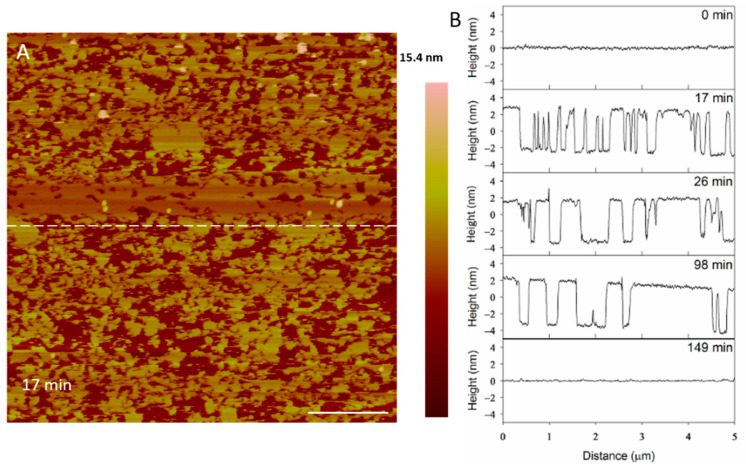
Evolution of PC membrane defects over time after exposure to 5 µM indolicidin. The dashed line (**A**) shows the location of the height profiles (**B**) on the substrate. The scale bar represents 1 µm.

The filling of the defects can also be observed in height profiles in Figure 9. The membrane defects may have been replaced with lipids, peptides or both. The end result was a smooth surface that appeared to be the same height as the original bilayer, suggesting that the material used to seal the defects is a lipid bilayer, rather than peptide–lipid aggregates, which may not have resulted in the same height. The shrinking “pores” shown at 98 min maintained a relative height of 5 nm (the height of a PC bilayer), suggesting that the resulting membrane at 149 min was a lipid bilayer.

**Figure 10:**
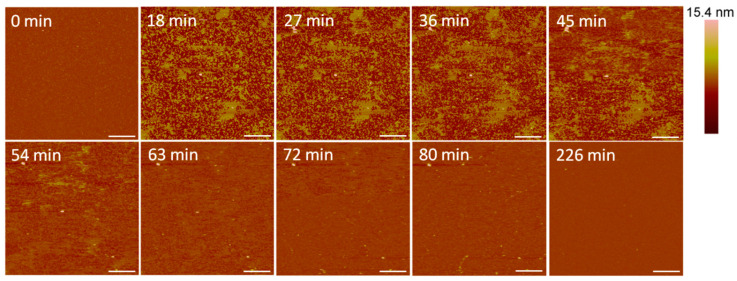
Defects formed in a mica-supported PC bilayer after exposure to 10 µM indolicidin. The scale bars represent a distance of 1 µm.

Small holes and channels were formed in the membrane at 10 µM concentrations (Figure 10). These defects left smaller patches of interspersed lipid bilayer membranes than 5 µM indolicidin. These defects also appeared to fill in over ~3.5 h, although the holes did not remain as well-defined as they were with 5 µM indolicidin during this process. While holes in the membrane were clearly differentiated from the lipid bilayer at 98 min in Figure 9, the AFM tip could not resolve the boundary between the lipid membrane and mica at 54 min in Figure 10. One possible reason for these “blurry” edges may have been that the lipids were not well attached to the mica and were moved by the force AFM tip as it scanned across the substrate surface.

**Figure 11:**
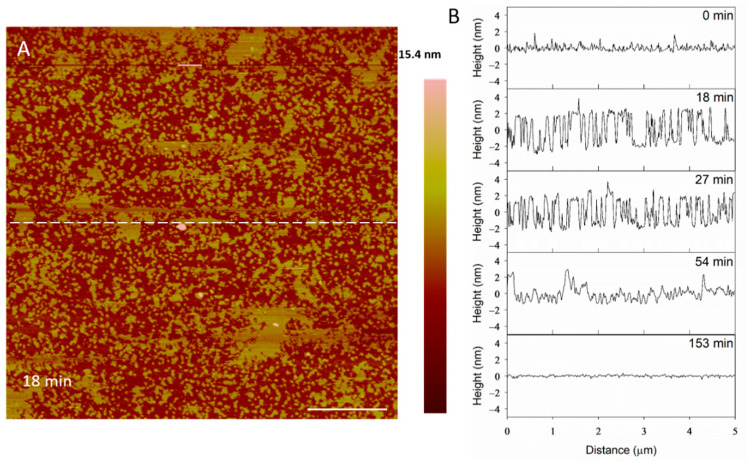
Filling of PC membrane defects over time with 10 µM indolicidin. The dashed line (**A**) shows the location of the height profiles (**B**). The scale bar is 1 µm.

The height profiles in Figure 11 show that the membrane defects that had formed in the bilayer 18 min after peptide exposure were 5 nm deep, which was expected in a 5-nanometer-tall lipid bilayer. After 27 min, however, the depth of these defects decreased to 4 nm. Within 54 min, the defect heights decreased to 3 nm, leading to a smooth surface at 153 min. The initial 5-nanometer defects that were captured at 18 min may correspond with the temporary complete insertion of the peptide into the membrane, but we cannot confirm or deny this hypothesis using these AFM images alone.

**Figure 12:**
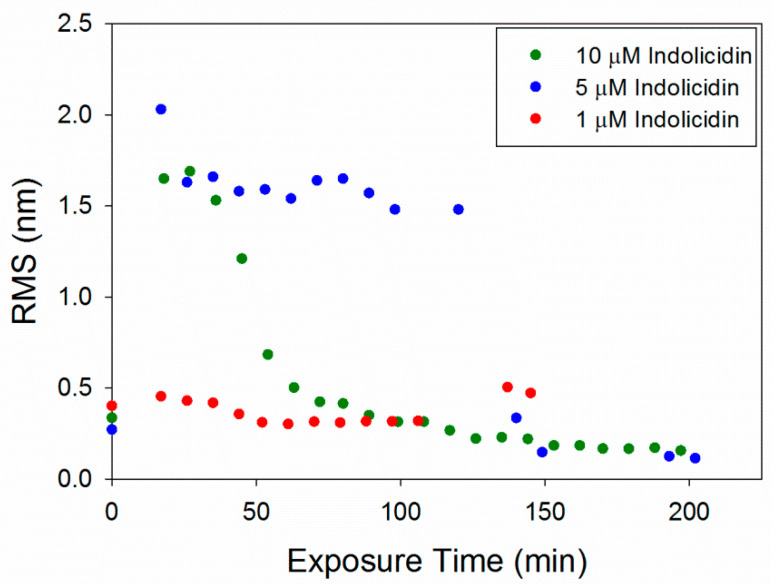
Root mean square (RMS) roughness values calculated for each 5 µm × 5 µm image of the supported PC bilayer after exposure to various indolicidin concentrations.

The RMS roughness plots in Figure 12 show that not much change occurred in the membrane with 1 µM indolicidin. The roughness of the membrane exposed to 5 µM indolicidin peaked at or before 17 min. Then, as the defects reorganized and filled in, the roughness decreased to around 1.6 nm until 3 h after peptide exposure, when the majority of the membrane became smooth. The roughness of the membrane when exposed to 10 µM indolicidin peaked at 27 min and quickly decreased as the membrane filled in. A similar trend was seen in an AFM study by Askou et al. showing the effect of 25 µM indolicidin on a supported PC membrane [23]. A plot of the average height roughness of the images over time revealed a peak in roughness between 15 and 30 min, which was followed by a gradual decrease in roughness before stabilizing. This common trend indicates that at these concentrations, the indolicidin-induced membrane damage peaks, at most, 30 min after exposure to the peptide. Therefore, the maximum amount of damage to a cell membrane may be observed within this time period when incubated with indolicidin.

The RMS values for 1 µM indolicidin (Figure 12) are about two times larger than those for 1 µM alamethicin (Figure 6) due to the presence of particles originally on the surface of the membrane before indolicidin was injected. More particles (possibly peptide aggregates) also attached to the membrane surface after 2 h of incubation with 1 µM indolicidin, resulting in the increased RMS values at ~140 min. The roughness values calculated for alamethicin-induced defects at 5 µM and indolicidin-induced defects at 5 and 10 µM also showed that both peptides substantially changed the lipid membrane surface within the first 50 min of exposure and gradually stabilized within ~150 min.

The indolicidin-induced membrane disruption captured using AFM imaging in this study complements and elucidates the peptide–membrane interactions observed in a previous QCM-D study [7]. The lack of visible defects in the PC membrane after exposure to 1 µM indolicidin (Figure 7) was consistent with the QCM-D results, which showed little change to the mass and lipid organization of the membrane after a 1-h incubation.

The loss of lipid mass from within the membrane at 5 µM indolicidin (Figure 8) was also observed in QCM-D experiments using the same peptide–lipid system [7]. The QCM-D measurements showed that approximately 10% of the lipid bilayer mass was removed from the substrate surface, which is similar to the amount of lipid removal observed in the AFM images at 62 min. However, the QCM-D results also showed a small amount of mass gain in the peptide mass on the membrane surface, which the AFM tip may not have been able to capture due to the nanometer-scale size of individual peptide molecules.

At 10 µM indolicidin, the incomplete filling of the membrane defects at 54 min (Figure 10) may be explained by partial insertion of the peptide that was observed in previous QCM-D experiments [7]. The QCM-D results indicated that indolicidin adsorbed to the surface of the PC membrane and partially inserted into membrane space where lipids had been removed. The mechanistic model that was derived from QCM-D measurements showed indolicidin partially inserting into a space where the top layer of lipids had been removed and the peptide associating with the remaining monolayer of lipids. If accurate, this mechanistic model would be consistent with the 3-nanometer defects (roughly half the height of the membrane) observed using AFM. The QCM-D results also indicated that the lipids within the membrane had become very disordered when exposed to 10 µM indolicidin, which may explain the “blurry” lipid patch edges that were captured in Figure 10.

The smoothing of peptide-induced defects in the lipid bilayer that was observed with 5 and 10 µM indolicidin has also appeared in several other AFM studies on indolicidin. Ha et al. found that ~15.7 µM indolicidin produced holes in mica-supported membranes (30–100 nm in diameter) composed of the synthetic lipid l-α-dipalmitoylphosphatidic acid (DPPA) that became smooth over time [43]. The membrane smoothing was thought to be due to the incorporation of peptides into the bilayer. Shaw et al. also imaged large, transient defects in the fluid domains of DOPC/DSPC membranes that disappeared slowly over time, which was hypothesized to be due to the fluidity of the membranes [29].

The concentration-dependence of the peptide–membrane interactions shown in this study is similar to the results obtained by Végh et al. on the effect of indolicidin on DPPC membranes formed on polyelectrolyte films on mica [30]. The polyelectrolyte film was present to create space for membrane-penetrating peptides between the bilayer and mica surface. As in our study, no membrane alterations were detected at low indolicidin concentrations (0.52 µM); though at higher concentrations (2.6 and 5.2 µM), the membrane remained intact, but particles appeared on the membrane surface. These particles were believed to be aggregates from excess amounts of indolicidin. This phenomenon also appeared in the AFM characterization of membranes exposed to 1 µM indolicidin. In a study by Végh et al., the DPPC bilayer structure did not form detectable membrane defects before 50 min of exposure to 15.7 µM indolicidin. The membrane was completely destroyed after 140 min of incubation with 7.9 and 15.7 µM indolicidin, but the smoothing of the membrane defects was not observed, as in our study. These differences were likely due to the variations in lipid membrane composition, as well as the presence of a polyelectrolyte film between the lipid membrane and mica surface. The polyelectrolytes may have inhibited the filling of defects by lipid or peptide material.

A concentration dependence of the effect of indolicidin on supported lipid bilayers was also reported by Nielsen et al., and their studies did not show any pore formation at concentrations ranging from 1 to 10 μm of indolicidin [19]. The authors used AFM, QCM-D, neutron reflectometry and small-angle X-ray scattering to examine the indolicidin–bilayer interactions, and they concluded that the interactions were at a surface level. In summary, the available studies show that indolicidin does not cause pore formation in the same manner that can be observed for some AMPs, but membrane perturbations at the surface can be observed, and they are concentration dependent. Whether the membrane can repair will depend on the concentration and time of the AMP exposure.

## 4. Conclusions

AFM imaging of AMP-induced changes in supported membranes is a valuable tool for the visual analysis of lipid removal and the stability of defects. While other techniques, such as QCM-D and oriented circular dichroism, also provide information on molecular-scale interactions, direct visual confirmation through AFM imaging is supportive to those analyses. Using AFM to examine the alamethicin- and indolicidin-induced defects in supported PC membranes revealed two distinct membrane-disruption mechanisms. The AFM images showed that 1 µM alamethicin did not substantially disturb the lipid bilayer, but higher alamethicin concentrations (5 and 10 µM) caused substantial a loss of lipids from the membrane. The membrane defects formed after exposure to 5 µM indolicidin continued to expand, causing the complete destruction of the lipid bilayer. At higher indolicidin concentrations (5 and 10 µM), AFM demonstrated smaller defects in the supported lipid bilayer, but they were able to correct themselves and smooth out over longer time scales. The examples of AMP-induced membrane disruption observed in this study offer insight into how various concentrations of AMPs may interact with and disrupt cell membranes. A fundamental understanding of the molecular mechanisms and dynamics associated with AMP–membrane interactions is instrumental for the selection and design of AMPs for applications in therapeutics.

## Data Availability

The data presented in this study are available within the article itself.
